# Late cutaneous metastases to the face from malignant pleural mesothelioma: A case report and review of the literature

**DOI:** 10.1186/1477-7819-7-84

**Published:** 2009-11-09

**Authors:** Alaaeldeen M Elbahaie, Dia E Kamel, Julia Lawrence, Neville G Davidson

**Affiliations:** 1 Clinical Oncology Department, Mid Essex Hospital Services NHS Trust, Broomfield Hospital, Court Road, Chelmsford, CM1 7ET, UK; 2 Histopathology Department, Mid Essex Hospital Services NHS Trust, Broomfield Hospital, Court Road, Chelmsford, CM1 7ET, UK; 3 Radiotherapy Department, Colchester Hospital University NHS Foundation Trust, Essex County Hospital, Lexden Road, Colchester, CO3 3NB, UK

## Abstract

**Background:**

Malignant Mesothelioma is a rare primary neoplasm affecting the serosal membranes. During its relative short course, this malignant neoplasm can give local and, rarely, distant haematogenous metastases in different organs. The reported metastatic sites include liver, lung, heart, brain, thyroid, adrenals, kidneys, pancreas, bone, soft tissue, skin and lymph nodes.

**Case Presentation:**

We report a sixty one year-old man with a history of malignant pleural epithelioid mesothelioma treated with six cycles of Pemetrexed and Carboplatin completed 03/11/04 followed by radiotherapy to the drain site 250 Kv/TD20Gy/5F completed 13/12/2004. Then he developed multiple facial skin lesions 4 years later. These lesions were proved to be metastatic malignant sarcomatoid mesothelioma.

**Conclusion:**

Mesothelioma metastases should be suspected in any known Mesothelioma patient with newly developed skin lesion.

## Background

Malignant Mesothelioma is a rare primary neoplasm affecting the serosal membranes. During its relative short course, this malignant neoplasm can give local and, rarely, distant haematogenous metastases in different organs. The reported metastatic sites include liver, lung, heart, brain, thyroid, adrenals, kidneys, pancreas, bone, soft tissue, skin and lymph nodes. The increased incidence of malignant mesothelioma and the improvement of survival rates due to the newly introduced chemotherapeutic agents bring to light the importance of studying its amended natural history.

## Case Presentation

A 61 year-old white man with known history of pleural mesothelioma on regular follow up was found to develop multiple facial skin lesions with no clinical evidence of local recurrence 4 years after the primary diagnosis.

**Figure 1:**
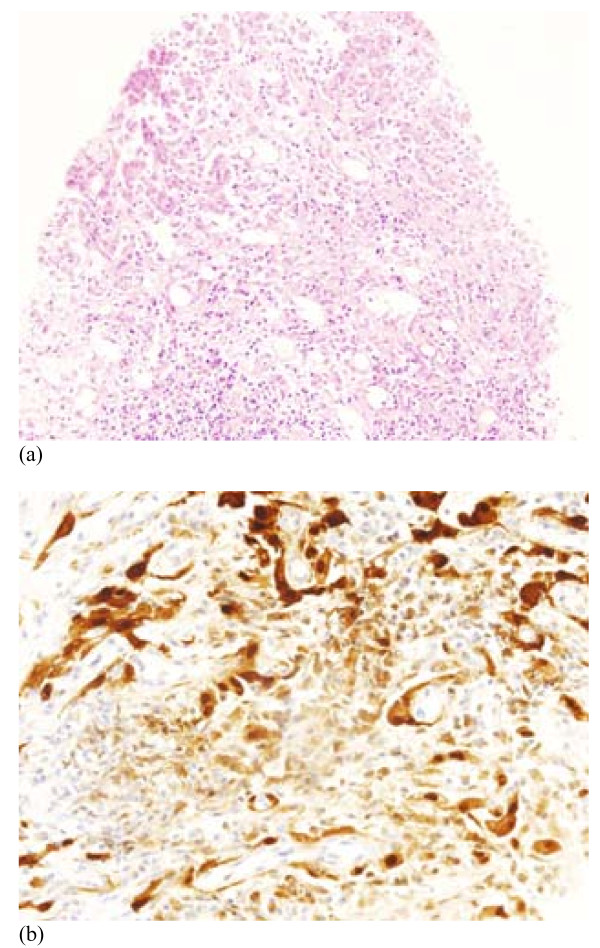
**Original pleural biopsy on June 2004, showing infiltration by epithelioid tumour cells (a), which are positive with immunohistochemical staining for Calretinin (b), consistent with epithelioid mesothelioma**.

On March 2004, this non-smoker, semi-retired boat builder with significant asbestos exposure history, presented with 4 months history of progressive shortness of breath. The inhalers had not helped and this seemed to be clearly a different problem to his original asthma. He also complained of some right chest pain, easy fatigue, dry cough and weight loss. On examination, there were only signs of pleural effusion. The chest X-rays showed increasing right pleural effusion and CT chest showed a large right simple pleural effusion with no pleural thickening or masses. He was admitted and the effusion drained. The cytology of the effusion was highly suggestive of mesothelioma, but pleural biopsy was insufficient. Few weeks later, the pleural effusion recurred and an US guided biopsy on 08/06/04 showed features consistent with a malignant Mesothelioma, epithelioid type (Fig. 1). The biopsy contains some skeletal muscle and some pleura with a thick layer of malignant epithelioid cells which are positive for mesothelial markers CK5/6 and Calretinin and negative for lung cancer markers TTF-1 and CEA.

The patient took part on the ALIMTA trial, he received 6 cycles of Pemetrexed 500 mg/m^2 ^+ Carboplatin AUC 5 day 1 every 3 weeks; the last cycle date was 03/11/04. This was followed by radiotherapy to the drain site "250 Kv/TD20 Gy/5F" completed 13/12/2004.

**Figure 2:**
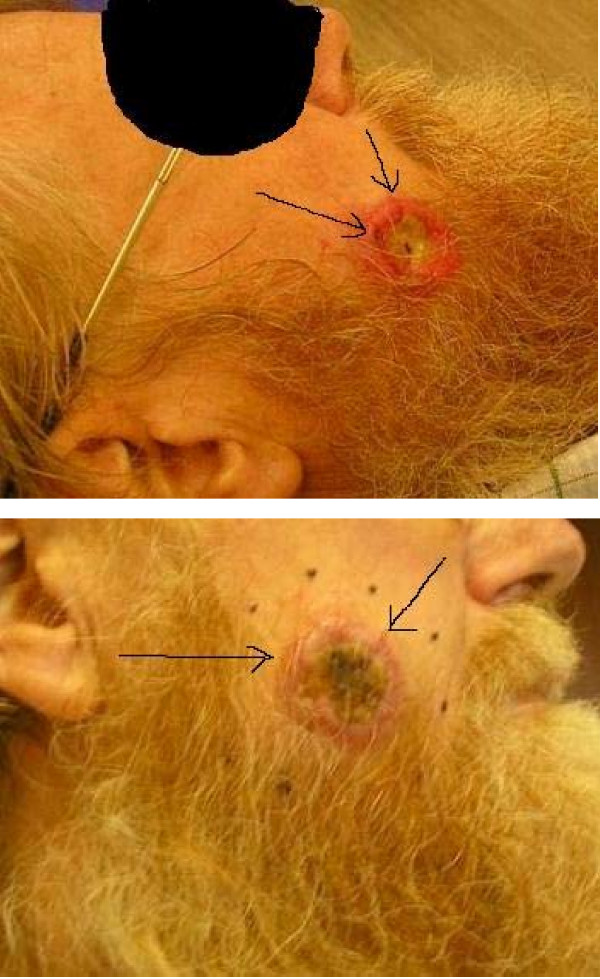
**Photos of skin lesion in the right cheek**.

Then, the patient underwent close follow up and he remained well and asymptomatic with no clinical or radiological evidence of disease recurrence until the end of December 2007, when he noticed small subcutaneous lesion on his right check and some nasal symptoms. Few weeks later, he developed fever 38°C with dry cough and the cheek lesion increased in size. CT scan on 17/03/2008 recorded several sites of disease; notably in the right hemithorax and right para-renal space consistent with recurrence of the Mesothelioma. Clinical Examination of the face revealed 3 skin lesions: right cheek 24 × 24 mm annular raised red area with eroded central area (Fig. 2), a small erythematous plaque posterior to the right ear and a 1 cm very firm subcutaneous lesion on the frontal area.

CT guided core biopsy from the right posterior basal pleural mass showed a thick layer of malignant epithelioid cells. A panel of immunohistochemical markers were performed and these malignant cells are positive for mesothelial markers CK5/6 and Calretinin and negative for lung cancer markers TTF-1 and CEA. Overall, these features are consistent with a malignant Mesothelioma, Epithelioid type.

**Figure 3:**
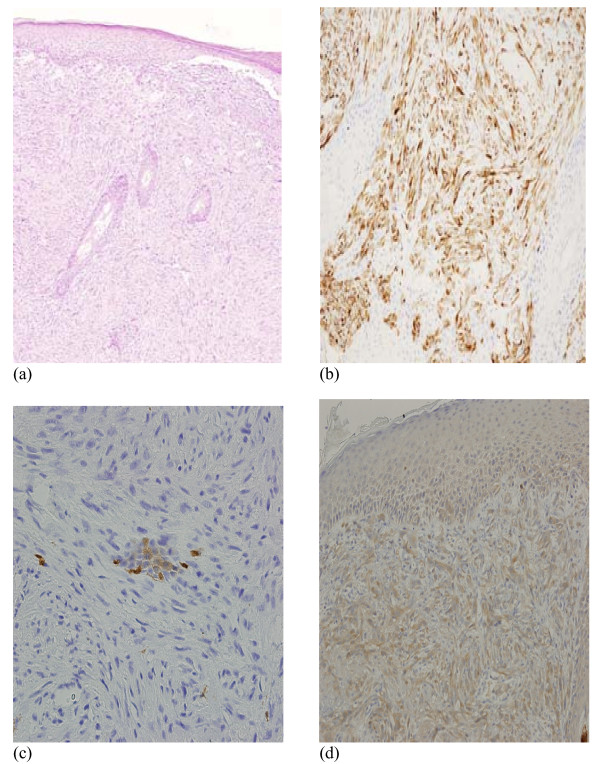
**Skin biopsy on May 2008 showing dermal infiltration by a spindle cell tumour (a), which was positive with cytokeratin CAM5.2 immunohistochemical staining (b), and focally positive with Calretinin (c) and mesothelin (d) consistent with sarcomatoid mesothelioma**.

The three skin lesions were biopsied. The microscopic examination showed sarcomatous atypical spindle cell proliferation within the dermis extending into the subcutaneous adipose tissue (Fig. 3). Mitotic figures including atypical forms are noted. The architecture of the tumour is partly storiform. Immunohistochemistry showed positivity with Vimentin, CAM 5.2 and CD10 and focally with SMA and Calretinin. The histopathological diagnosis was in keeping with Metastatic Mesothelioma of sarcomatous type.

## Discussion

Malignant Mesothelioma is a rare primary neoplasm affecting the serosal membranes with a relative increase of its incidence rate during the last decades [1]. Most cases of mesotheliomas are related to asbestos exposure [2]. Its incidence has been rising steadily over the past few decades [1]. Approximately 1000 people die of mesothelioma each year in the UK, and it is predicted to rise to 3000 by the year 2020 [3].

Histologically, Mesothelioma is divided into epithelial, sarcomatous and mixed or biphasic subtypes. In several series epithelial type has a significantly improved prognosis compared to sarcomatous variant [4]. The primary diagnosis of our patient was epithelioid type; then the local recurrence was also epithelioid, while the skin metastases are of sarcomatous type; which may be explained by the heterogeneous nature of the disease or the known fact that malignant cells may loose some of there differentiation when metastasis.

Systemic therapy is the only treatment option for the majority of mesothelioma patients. For many years, chemotherapy had a minimal impact on the natural history of this cancer. Countless drugs were evaluated, most of which achieved response rates below 20% and median survival of <1 year [5]. In recent years, there has been a surge of optimism regarding systemic treatment of this disease. Several cytotoxic agents have been shown to generate reproducible responses, improve quality of life, or prolong survival in mesothelioma. Drugs with single-agent activity include pemetrexed, raltitrexed, vinorelbine, and vinflunine [5]. The combination of pemetrexed plus cisplatin is considered the benchmark front-line regimen for this disease, based on a phase III trial in 456 patients that yielded a response rate of 41% and a median survival of 12.1 months compared to 9.3 months for single agent cisplatin [6]. A recent large International Expanded Access Program confirmed the activity of pemetrexed plus cisplatin and pemetrexed plus carboplatin in chemonaive patients with Malignant Pleural Mesothelioma, demonstrating clinically similar time to progressive disease and 1-year survival rates [7]. Our patient received 6 cycles of Pemetrexed 500 mg/m^2 ^+Carboplatin AUC 5 day 1 every 21 days, completed 03/11/04 with >37 months disease free survival. This long disease free survival in mesothelioma patients is rare; however this may be explained by the small disease burden on primary presentation and/or treatment received.

During its relative short course, this malignant neoplasm, independently of the therapy, can give local or distant haematogenous metastases in different organs. The reported metastatic sites include liver, lung, heart, brain, thyroid, adrenals, kidneys, pancreas, bone, soft tissue, skin and lymph nodes [8,9].

Only small number of cases of subcutaneous metastases of malignant Mesothelioma has been reported. However, the majority of the reported cases were considered as local invasion of the disease. To our knowledge, in English language published articles, there are only 10 reported cases of pleural Mesothelioma with distant subcutaneous metastases [8,10-17]; seven of them had metastases to the face and/or scalp. So, our case is the 11^th ^reported pleural Mesothelioma case with a distant subcutaneous metastasis and it is the 8^th ^case with face and/or scalp subcutaneous metastases [8,10-14].

## Conclusion

With the increased incidence of malignant Mesothelioma and the improvement of survival rates due to the newly introduced chemotherapeutic agents, the number of recorded distant skin metastases is likely to increase. Metastatic disease from mesothelioma should be suspected in any known mesothelioma patient who develops a new malignant skin lesion.

## Competing interests

The authors declare that they have no competing interests.

## Authors' contributions

AE is the main author; he did a major part in the clinical work, all the literature review, all the editing work and publication submission. ND is the head of the department who supervised all the steps of the work and his invaluable advices were essential in finalizing the article. DK did the histopathological work. JL shared in the clinical work.

## Consent statement

Written informed consent was obtained from the patient's next of kin (his widow; as the patient is deceased) for publication of this case report and accompanying images. A copy of the written consent is available for review by the Editor-in-Chief of this journal.
